# Chemoradiation provides a physiological selective pressure that increases the expansion of aberrant *TP53* tumor variants in residual rectal cancerous regions

**DOI:** 10.18632/oncotarget.2438

**Published:** 2014-10-07

**Authors:** Kazuko Sakai, Shinsuke Kazama, Yuzo Nagai, Koji Murono, Toshiaki Tanaka, Soichiro Ishihara, Eiji Sunami, Shuta Tomida, Kazuto Nishio, Toshiaki Watanabe

**Affiliations:** ^1^ Department of Genome Biology, Kinki University Faculty of Medicine, Osaka-Sayama, Osaka, 589-8511, Japan; ^2^ Department of Surgical Oncology, The University of Tokyo, Bunkyo-ku, Tokyo, 113-8655, Japan

**Keywords:** Chemoradiation, Rectal cancer, *TP53*, deep sequencing, RNA sequencing

## Abstract

Neoadjuvant chemoradiotherapy has been introduced in patients with surgically resected rectal cancer and reduced the local recurrence. Heterogeneity exists in rectal cancer, and we hypothesized that there are subclones resistant to chemoradiotherapy within the cancer mass.

We performed DNA-targeted sequencing of pre- and post-treatment tumor tissues obtained from 20 rectal cancer patients who received chemoradiotherapy. The variant frequency of the mutant clones was compared between pre- and post-treatment samples of nine non-responder patients. RNA-targeted sequencing of 57 genes related to sensitivity to chemotherapy and radiotherapy was performed for the paired samples. Immunohistochemical analyses of p53 expression were also performed on the paired samples from the nine non-responder patients.

DNA-sequencing detected frequent mutations of suppressor genes including *TP53*, *APC* and *FBXW7* in the post-treatment samples of the nine non-responders. The frequency of *TP53* mutations showed significant increases after chemoradiotherapy. RNA-targeted sequencing of 29 tumor tissues demonstrated that decreased expression of three genes and increased expression of four genes were detected in the post-treatment samples. Significantly increased expression of *TP53* was observed in the post-treatment samples. Immunohistochemical staining for p53 revealed that increased p53 intensity scores were observed after chemoradiotherapy. These results suggest that the tumors with *TP53* mutations tend to accumulate through chemoradiotherapy.

## INTRODUCTION

Approximately 1.2 million people are diagnosed with colorectal cancer (CRC) each year, and ~600,000 people die from the disease worldwide [1]. Surgical treatment is performed to cure the disease, however, various types of recurrence develop after surgery. Among these, local recurrence is observed more frequently in rectal cancer than colon cancer patients, and frequently impairs the patient's quality of life [2, 3]. Therefore, pre-operative chemoradiotherapy has been introduced as neoadjuvant therapy in the treatment of rectal cancer in order to reduce the local recurrence rate [4, 5]. However, patients with rectal cancer exhibit a wide spectrum of responses to chemoradiotherapy. About 10% of the patients who receive chemoradiotherapy achieve a pathological complete responses (pCR), while one-third die within five years [2, 3]. It is important to increase the efficacy of chemoradiotherapy and improve patient outcomes. Antimetabolite is a major chemotherapeutic agent used in combination with radiotherapy. However, other new agents have also been active when used in combination with radiotherapy for patients with rectal cancer. The clinical question remains, which chemotherapeutic would be best for use in combination with radiotherapy. Deep sequencing might give us the answer to this question. Therefore, the biomarkers predicting the sensitivity of chemoradiotherapy are clinically important.

Microarrays have been used extensively to identify differential gene expression profiles in many cancers, which has aided in the screening, prognosis and classification of tumors. There have been a few previous studies that have reported a signature of gene expression classifying the sensitivity of the particular tumor to chemoradiotherapy [6, 7]. However, microarrays have limitations, including their low sensitivity [8, 9], low dynamic range and the possible presence of hybridization artifacts [10]. In addition, relatively large amounts of frozen tumor tissue samples are necessary for this technology.

Recent cancer profiling studies have focused on next-generation sequencing (NGS) [11]. RNA sequencing of the steady-state RNA expression avoids the limitations of microarray expression and allows for massive parallel sequencing of millions of sequences on chips containing complementary DNA (cDNA) libraries, generating a higher number of transcript sequences than is possible by a microarray analysis [12]. Thus, RNA sequencing presents unprecedented possibilities for genomic characterization, and has significantly advanced our understanding of genomic organization, including allele-specific expression, novel transcripts and variant isoforms. The advantages of RNA sequencing include its large dynamic range, high sensitivity and rapid sequencing for the differential expression analysis [12].

It has often been discussed that the expression data for more than 20,000 genes is associated with high redundancy, and a large sample size is necessary for this type of validation. Therefore, in this study, we aimed to analyze the gene expression profiles of 57 selected genes. This gene set was selected based on the biological evidence of the chemosensitivity of tumors to antimetabolites and platinum agents, and the radiosensitivity of tumors (Supplementary Table 1). Some of the oncogene and suppressor genes were selected because these genes were reported to be related with sensitivity to cytotoxic chemotherapy and radiotherapy. In addition, family of topoisomerases (TOP1, TOP2A, and TOP2B) and uridine diphosphate (UDP)-glucuronosyl transferase 1A1 (UGT1A1) were included into the set because the topoisomerase inhibitor CPT-11 might be used for chemoradiotherapy. Tubulin beta 3 (TUBB3) and vascular endothelial growth factor (VEGFA) were included as possible markers for antimitotic agents and anti-angiogenesis inhibitors. NGS is also a widely-used technology for the gene analysis. However, the use of targeted RNA sequencing using the NGS technology has been limited for clinical formalin-fixed paraffin-embedded (FFPE) samples. In this study, we set up a multiple gene expression analysis for FFPE samples using targeted RNA sequencing (Ion-PGM, Life Technologies, Carlsbad, CA).

The development of CRC is a multistep process that involves the accumulation of a wide range of genetic and phenotypic alterations, leading to the aberrant expression of genes that regulate cell proliferation. Therefore, somatic mutations are often detected in CRC, and these mutations are part of key mechanisms resulting in carcinogenesis. Somatic mutations of cancer-related genes are also related to the chemoradiosensitivity and resistance of solid tumors. For example, the *KRAS* mutation status is related to chemoradioresistance [13], as well as treatment with anti-EGFR antibodies [14]. However, there have been no previous studies comparing the mutation profiles of the tumors and the pre- and post-treatment chemoradiosensitivity.

*TP53* is an integral part of the DNA damage-induced apoptosis pathway, and mutations and overexpression of *TP53* are frequently observed in rectal cancer. Regions of low oxygen and necrosis are common features of solid tumors. We hypothesized that chemoradiation provides a physiological selective pressure on tumors that induces the expansion of variant cells, leading to the acquisition of *TP53* mutations. To test this hypothesis, we performed targeted sequencing for clinical FFPE specimens obtained from rectal cancer patients, and we tried to compare the mutation profiles of the tumor samples pre- and post-treatment, focusing on the *TP53* tumor-suppressor gene.

## RESULTS

### Somatic mutation profiling by targeted DNA sequencing

**Figure 1:**
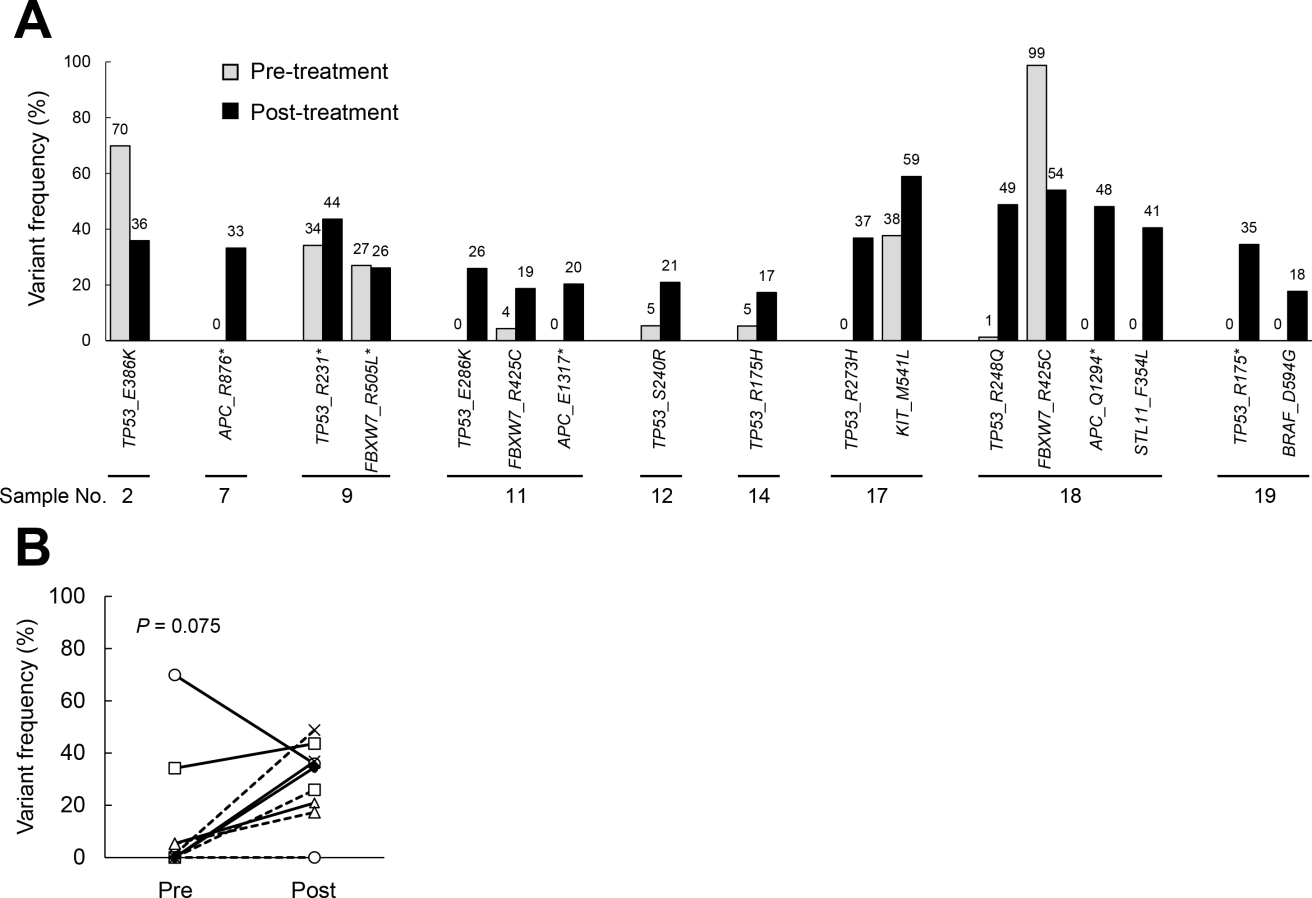
The frequency of gene mutations in samples obtained pre- and post-chemoradiation The gene mutation status was evaluated by DNA sequencing, and the frequencies were compared between paired pre- and post-treatment samples from nine non-responders. **(A)**. The mutation frequency in the pre- and post-chemoradiotherapy tumors. **(B)**. The changes in the frequency of *TP53* mutations in the pre- and post-chemoradiation samples. Paired t-tests were used for the statistical analysis.

Among the 20 patients, 10 were classified as responders and 10 as non-responders, according to the histopathological examination. After chemoradiotherapy, responders and non-responders represented with pathological grade 2 and 1a, respectively (Supplementary Table 2). Median disease-free survival (DFS) and overall survival (OS) of total patients were 61.4 and 71.4 months, respectively (Supplementary Figure 1A). These are comparable to that in our previous reports [15]. In comparing DFS and OS between responder and non-responder group, median DFS (69.7 versus 55.9 months, *P* = 0.0560) and OS (79.9 versus 62.8 months, *P* = 0.0263) were longer in responders than non-responders (Supplementary Figure 1B and 1C). Somatic mutation profiling was performed by the targeted DNA sequencing of 50 cancer-related genes in the paired pre- and post-treatment samples from nine of the non-responders, because a sufficient amount of tumor cells were not obtained in the post-treatment tissues in one case (No. 8). Among the post-treatment samples of these nine non-responders, we detected *TP53* mutations in eight cases (89%), *APC* mutations in three cases (33%), *FBXW7* mutations in three cases (33%), and *STK11*, *KIT* and *BRAF* mutations in one case each (11%). The frequency of corresponding mutations in the pre-treatment samples was analyzed. When comparing the pre- and post-treatment samples in each pair, we found that there was an increased frequency of mutants of the tumor suppressor genes in the post-treatment samples (Figure 1A). The frequency of *TP53* mutations tended to increase in the post-treatment samples compared to the pre-treatment samples (*P* = 0.0752, by a paired t-test, Figure 1B and Table 1). This result led us to surmise that the accumulation of mutations of tumor suppressor genes may be increased by chemoradiotherapy.

**Table 1:** 

ID	*TP53* mutation site	*TP53* mutant frequency (%)		*TP53* relative gene expression		p53 IHC scoring index	
		pre	post	pre	post	pre	post
2	*TP53_E386K*	69.9	35.9	5776	6105	70	160
7	no *TP53* mutation	-	-	4182	2445	100	100
8	n.t.	n.t.	n.t.	n.t.	n.t.	n.t.	n.t.
9	TP53_R231*	34.2	43.6	764	n.d.	0	50
11	*TP53_E286K*	0	25.9	0	8926	75	160
12	*TP53_S240R*	5.37	20.9	3482	7052	75	75
14	*TP53_R175H*	5.26	17.3	1878	7879	70	100
17	*TP53_R273H*	0	36.8	4314	10663	160	160
18	*TP53_R248Q*	1.26	48.8	1776	9247	140	180
19	*TP53_R175H*	0	34.5	5802	n.d.	55	180

n.t., a sufficient amount of tumor cells were not obtained in the post-treatment tissues; n.d., not detected.

### Changes in gene expression before and after chemoradiotherapy, as determined by RNA sequencing

We also performed targeted RNA sequencing on 29 clinical FFPE samples (including nine paired non-responder samples, 1 pre-treatment non-responder sample and 10 pre-treatment responder samples) obtained from the rectal cancer patients. Twenty nanogram RNA samples were subjected to a gene expression analysis by RNA sequencing for the 57 targeted genes. The median coverage of each sample ranged from six to 4019. Two post-treatment samples (Nos. 9 and 19) showed extremely low median coverage (six and 28, respectively), and these were filtered out from the further analysis. The geometric mean was calculated and used for normalization. The levels of expression of each gene in the responder and non-responder groups were plotted (Supplementary Figure 2). A non-parametric statistical method (Mann-Whitney U-test) was used for comparisons between the responders and non-responders.

The expression of *ABCG2* (*P* = 0.0196) and *TYMP* (*P* = 0.0343) was decreased in non-responders compared with responders. In contrast, increased expression of *NRP1* (*P* = 0.0343), *RPN2* (*P* = 0.0233), *TOP1* (*P* = 0.0413) and *TYMS* (*P* = 0.0343) was observed in non-responders compared with responders. The expression of tumor suppressor genes, including *TP53*, *PTEN* and *STK11*, was not significantly different between the two groups.

Next, the changes in gene expression between pre- and post-treatment samples in the non-responder group were plotted (Supplementary Figure 3). A paired t-test was used for comparisons between these pre- and post-treatment samples. Increased expression of *ABCG2* (*P* = 0.0196), *GSTP1* (*P* = 0.0177), *HMGB2* (*P* = 0.0259), *STMN1* (*P* = 0.0006), *TP53* (*P* = 0.0241), *TUBB3* (*P* = 0.0226) and *TYMP* (*P* = 0.0022) was observed in the post-treatment samples compared to the pre-treatment samples. In contrast, decreased expression of *DPYD* (*P* = 0.0181), *ERCC3* (*P* = 0.0056), *RPN2* (*P* = 0.0105), *RPM1* (*P* = 0.0453) and *TOP1* (*P* = 0.0447) was detected in the post-treatment samples.

**Figure 2:**
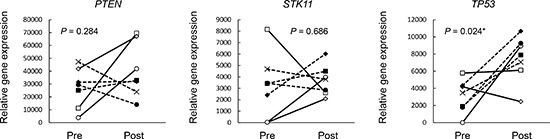
The results of the gene expression analysis of paired non-responder tumor samples The gene expression levels were evaluated by RNA sequencing, and the expression was compared in paired pre- and post-chemoradiotherapy samples from nine non-responders. The expression of three major tumors suppressor genes (*PTEN*, *STK11* and *TP53*) in paired samples is shown. Paired t-tests were used for the statistical analysis.

To evaluate our hypothesis, we focused on the gene expression changes in tumor suppressor genes (*TP53*, *PTEN*, and *STK11*) between the pre- and post-treatment samples. Increased expression of *TP53* was observed in the post-treatment samples (*P* = 0.024, Figure 2, Table 1, and Supplementary Table 3).

### Immunohistochemical staining of the p53 protein

**Figure 3:**
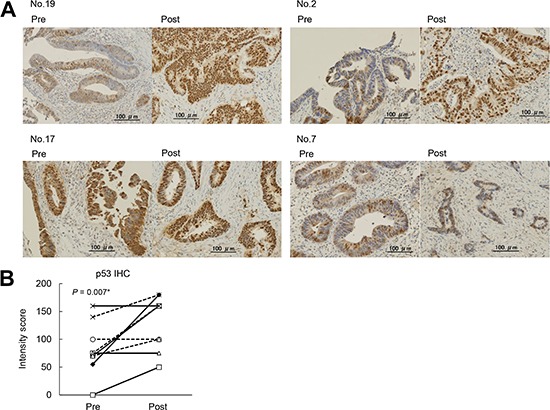
Immunohistochemical staining of paired tumor samples using an anti-p53 antibody **(A)**. Representative staining for p53 in paired non-responder samples. (original magnification, x25). No.19 and No.2 cases were increased p53 staining group. No.17 and No.7 cases were no change group. **(B)**. The scores for p53 staining were compared between pre- and post-chemoradiotherapy samples. The intensity score = (average of area (%) × intensity of staining (0-2). Paired t-tests were used for the statistical analysis.

To confirm our hypothesis, we performed immunohistochemical staining of p53 (the protein encoded by *TP53*) in paired samples using anti-p53 antibodies. Only nine samples were evaluable because there was no tumor tissue in one case. Increased p53 staining, as determined by the Intensity Score (area × intensity) for p53, was observed in six of the 9 post-treatment samples, and no change was observed in the other three samples (Figure 3, Table 1, and Supplementary Table 4). It is well known that overexpression of the p53 protein sometimes accompanies *TP53* mutation status (loss of function) [16]. Therefore, our results are consistent with the results of the DNA sequencing, and suggest that enrichment of tumor clones with alterations in tumor suppressor genes occurred during or after the chemoradiotherapy.

## DISCUSSION

In this report, the frequency of *TP53* mutations was significantly enriched in the post-treatment samples compared to the pre-treatment samples. It is well known that colon cancer exhibits high heterogeneity. It can therefore be speculated that *TP53* mutation-positive tumor cells are present in most tumors. Mutations in *TP53* occur in half of all human cancers [17], indicating its critical importance in inhibiting cancer development. *TP53* is involved in apoptosis, the response to DNA damage and cell cycle repair. The chemosensitivity and radiosensitivity of several types of cancers have been demonstrated to be *TP53*-dependent in the number of preclinical studies [16, 18-20]. Further, the presence of *TP53* mutations significantly decreased the radiation-induced senescence in head and neck squamous cell lines [19]. In addition, *TP53* mutations and overexpression were associated with the response to neoadjuvant treatment in breast cancer patients in the clinical setting [21]. Recently, the *TP53* genotype was reported to be associated with the survival after adjuvant treatment in female colon cancer patients in a clinical study (CALGB89803) [22].

We found that there was an increased population of tumors with *TP53* mutations and *TP53* overexpression in the post-treatment samples. When we compared the mutation profiles between the pre- and post-treatment samples, the same types of tumor suppressor gene mutations were detected (Figure 1). Based on that finding, we speculated that chemoradiation provides a physiological selective pressure for the expansion of *TP53* mutant tumor variants in residual cancerous tissue. An increased frequency of *TP53* mutants and an increased intensity of p53 immunostaining were consistently observed in post-treatment samples compared with the respective pre-treatment samples in five of the nine cases. No change in the *TP53* mutation frequency and p53 immunostaining were observed between the pre- and post-treatment samples in one case (No. 2). In the other two cases, a higher frequency of *TP53* mutations was observed in the pre-treatment samples compared to the post-treatment samples, whereas no differences were observed by p53 immunostaining.

On the other hand, it is not surprising that there was a discrepancy in the changes in the *TP53* status between pre- and post-treatment samples at the genome, transcript and protein expression levels, because *TP53* expression is regulated, at least partly, by post-translational regulation. In a series of 67 breast tumors, 19% had *TP53* gene mutations, 40% had a positive *TP53* IHC result and 12% had both [21].

In our study, we found an increased population of tumor cells with *FBXW7* mutations after chemoradiation in two of the three cases examined. *FBXW7* (also known as Fbw7, Sel-10, hCdc4 or hAgo), an F-box protein subunit of an SCF-type ubiquitin ligase complex, induces the degradation of positive regulators of the cell cycle, such as c-Myc, c-Jun, cyclin E and Notch. *FBXW7* is often mutated in a subset of human cancers including CRC [23, 24]. Thus, *FBXW7* is also a critical tumor suppressor gene. Because *FBXW7* also participates in the cell cycle exit to, and the reentry from, the G_0_ phase, it is a candidate molecular therapeutic target in intractable carcinoma cases that are resistant to combined modality therapies [25-27]. Onoyama *et al*. reported consecutive roles for *TP53* and *FBXW7* in the carcinogenesis of solid tumors *in vivo*. Moreover, a comparison of four groups classified according to the *FBXW7* and *TP53* status revealed a worse prognosis for double inactivation mice compared to the other subgroups [27]. The clinical significance of *FBXW7* in human solid cancers has been diversely reported, and lower expression of *FBXW7* was associated with chemoresistance in one study [28].

In our study, we also found an increased population of tumor cells with *APC* mutations after chemoradiation in all three cases examined. The adenomatous polyposis coli (APC) tumor suppressor is the most commonly mutated gene in colorectal cancers. In addition to its effects on the Wnt/β-catenin signaling pathway, APC also regulates other processes in a Wnt/β-catenin-independent manner, such as the apical-basal polarity, microtubule networks, cell cycle progression, DNA replication and repair, apoptosis and cell migration [29].

A limitation of the present study is the small sample size which may reduce the power of the study. Still, we were able to detect differences in the expression of some genes in samples before and after treatment, albeit, the data must be interpreted cautiously since it is difficult to arrive to a solid conclusion because of the limited sample size. Nevertheless, we have shown that in principle, gene expression analysis targeting RNA sequencing of FFPE samples is feasible using NGS.

In summary, the results from the present study suggest the possibility that chemoradiotherapy induces plasticity and therapeutic escape in cancer cells and is manifested by the differential expression of genes from resistant cancer cells present in residual colorectal tumors. The present sample set has allowed us to explore possible relationship of *TP53* between responders and non-responders for chemoradiotherapy and revealed differences in the mutation rates of various genes, including *TP53*. In order to strengthen our hypothesis, we included the data of gene expression, protein expression (IHC) from the same sample set. To conclude, this study provides the foundation to warrant future large scale studies to validate *TP53* alterations after chemoradiation in colorectal cancer patients.

## MATERIALS AND METHODS

### Patients and samples

In order to elucidate biological differences in response to chemoradiotherapy, we compared differences between an equivalent number of samples of responders and non-responder cases. A total of 20 rectal cancer patients who chose to receive preoperative chemoradiotherapy at the Department of Surgical Oncology, the University of Tokyo Hospital, between 2006 and 2011 were analyzed retrospectively. Biopsy samples were taken from the rectal cancers during colonoscopic examinations before preoperative chemoradiotherapy was performed, and these were classified as “pre-treatment samples”. All patients underwent surgical resection of the rectal cancer, and the surgically resected specimens were classified as “post-treatment samples”. Both the pre-treatment and post-treatment samples were fixed in 10% formalin and embedded in paraffin. Informed consent was obtained from all patients for the collection of specimens for future analyses, and the study protocol was approved by the Ethics Committee of Tokyo University Faculty of Medicine and the Kinki University Faculty of Medicine. The characteristics of the patients included in this study are summarized in Supplemental Table 2.

All patients received a total dose of 50.4 Gy of radiation, given in 28 fractions over six weeks. Tegafur-uracil (300–500 mg/day) and leucovorin (75 mg/day) were given concomitantly with radiotherapy. Standardized curative resection was performed six weeks after the completion of chemoradiotherapy. The response to chemoradiotherapy was determined by a histopathological examination of the surgically resected specimens based on a semiquantitative classification system defined by the Japanese Society for Cancer of the Colon and Rectum. In brief, grade 0 indicated no tumor cell necrosis or degeneration; grade 1 indicated tumor cell necrosis or degeneration in less than two-thirds of the entire lesion; grade 2 indicated prominent tumor cell necrosis or degeneration in more than two-thirds of the entire lesion, but with viable tumor cells remaining and grade 3 indicated that there were no viable tumor cells (Japanese Classification of Colorectal Carcinoma. Kanehara & Co., Ltd, Tokyo, Japan). Tumors were classified as “responders” when assigned regression grade 2 or 3, and “non-responders” when assigned grade 0 or 1. As noted above, the FFPE pre-treatment samples were obtained by biopsy. The post-treatment samples were obtained by surgical resection after chemo-radiation in non-responder cases.

### DNA and RNA extraction

Collected FFPE specimens were subjected to a histological review, and only those containing sufficient tumor cells (at least 75% tumor cells) as determined by hematoxylin and eosin (H&E) staining were subjected to DNA/RNA extraction. DNA and RNA were purified using an Allprep DNA/RNA FFPE kit (Qiagen, Valencia, CA) according to the manufacturer's instructions. The quality and quantity of the DNA/RNA were verified using the NanoDrop 2000 device (Thermo Scientific Wilmington, DE), PicoGreen dsDNA assay kit (Life Technologies) and the RiboGreen RNA assay kit (Life Technologies). The extracted DNA/RNA was stored at −80°C until the analysis.

### RNA sequencing

For RNA sequencing, PCR primers were designed using the Ion AmpliSeq Designer software program (Life Technologies). The Ion AmpliSeq RNA Library Kit (Life Technologies) was used to construct the RNA library according to the manufacturer's instructions. Briefly, 20 ng of total RNA were reverse transcribed with the SuperScript III enzyme, followed by PCR amplification. The Ion Xpress Barcode adapters (Life Technologies) were ligated into the PCR products and purified with Agencourt AMPure XP beads (Beckman Coulter, Brea, CA). Purified libraries were pooled and sequenced on an Ion Torrent PGM (Life Technologies) using the Ion PGM 200 Sequencing Kit v2 and the Ion 318 v2 Chip Kit.

### DNA sequencing

We used 20 ng of DNA for the multiplex PCR amplification using the Ion AmpliSeq Library Kit and the Ion AmpliSeq Cancer Hotspot Panel v2 (Life Technologies) according to the manufacturer's instructions. The Ion Xpress Barcode Adapters (Life Technologies) were ligated into the PCR products and purified with Agencourt AMPure XP beads (Beckman Coulter). Purified libraries were pooled and sequenced on an Ion Torrent PGM device (Life Technologies) using the Ion PGM 200 Sequencing Kit v2 and the Ion 318 v2 Chip Kit.

DNA sequencing data were accessed through the Torrent Suite v.3.4.2 software program. Reads were aligned against the hg19 human reference genome, and variants were called using the variant caller v 3.6. Raw variant calls were filtered out using the following annotations: homozygous and heterozygous variants, quality score of <100, depth of coverage <19.

### Immunohistochemical analysis

Immunohistochemical analyses were performed on nine paired samples from non-responder patients. Deparaffinized and rehydrated sections were heated in a microwave oven for seven three-minute cycles in citrate buffer to retrieve antigens, and were cooled for 15 min at room temperature. The endogenous peroxidase activity was inhibited by incubation of the samples with 0.3% hydrogen peroxidase in methanol for 20 min at room temperature. After blocking the non-specific reactions with 10% normal rabbit serum, the sections were first incubated with an anti-p53 antibody (mouse monoclonal antibody DO 7; Novocastra) overnight at a dilution of 1:100. The sections were then incubated with biotinylated rabbit anti-mouse immunoglobulin for 30 min, and next with streptavidin–peroxidase complex (Histofine SAB-PO Kit, Biogenex Laboratories) for 15 min. The sections were carefully rinsed with several changes of phosphate-buffered saline (PBS) between each step of the procedure. The color was developed with diaminobenzidine. The sections were lightly counterstained with hematoxylin and mounted. Negative controls were obtained by replacing the primary antibody with PBS. The specimens immunostained for p53 were independently evaluated by two authors (S.K and T.W) who received training of pathological diagnosis without knowledge of the clinicopathological features. Nuclear staining of cancer cells was evaluated according to both the stained area and the intensity of the nuclear-stained cancer cells observed under 25× magnification. We scored the intensity of the nuclear-stained cancer cells into 3 categories: none; 0, moderate; 1+, or strong; 2+, respectively.

### Statistical analysis

A non-parametric statistical method (Mann-Whitney U-test) was used for comparisons between the responders and non-responders. The paired t-test was used for comparisons between pre-treatment and post-treatment samples in the non-responders. Statistical analyses were carried out using the JMP software program (version 10; SAS Institute, Cary, NC). A value of *P* < 0.05 was considered to be statistically significant.

## SUPPLEMENTARY TABLES AND FIGURES


