# Supplementary material for: Chemoradiation provides a physiological selective pressure that increases the expansion of aberrant TP53 tumor variants in residual rectal cancerous regions
Source: Oncotarget. 2014 Oct 7;5(20):9641–9. doi: 10.18632/oncotarget.2438 (PMC4259426; doi:10.18632/oncotarget.2438)
Supplement: Supplementary file 1 [file oncotarget-05-9641-s001.pdf]

SUPPLEMENTARY TABLES AND FIGURES

Supplementary Table S1. Gene list for RNA sequencing. The genes were selected for RNA sequencing based on their involvement in the response to chemo- and radiotherapy

Gene list associated with chemoradiotherapy for RNA sequencing

DNA damaging agent and radiotherapy	Genes related to DNA repair	*ERCC1*
		ERCC2
		ERCC3
		ERCC6
		ERCC8
		XRCC1
		XRCC3
		OGG1
		RAD51
		RRM1
		TBP
	Suppressor genes	*BRCA1*
		PTEN
		STK11
		TP53
	Genes related to drug metabolism	*HMGB1*
		HMGB2
		MGMT
		GCLC
		GSTA1
		GSTP1
	Genes related to detoxication	*MT1A*
		MT2A
Antimetabolite	Genes related to folate metabolism	*DHFR*
		DPYD
		DUT
		FPGS
		MTHFR
		NP
		PRPS1
		TK1
		TYMP

(*Continued*)

Gene list associated with chemoradiotherapy for RNA sequencing

		TYMS
		UCK2
		UMPS
		UPP1
Topoisomerase inhibitor	Genes related to drug transporter	*ABCG2*
		ABCB1
	Genes related to drug metabolism	*CES1*
		CES2
		TOP1
		TOP2A
		TOP2B
		UGT1A1
Antimicrotubule agent	Oncogenes	*ERBB2*
		FLT1
		STMN1
	Genes related to microtubule	*MAPT*
		TUBB3
	Genes related to glycosyltransferase	*RPN2*
Antiangiogenesis agent	Genes related to angiogenesis	*ARNT*
		HIF1A
		KDR
		NRP1
		NRP2
		PROK2
		VEGFA

Supplementary Table S2. The clinical information regarding the 20 CRC patients

No.	Response to CRT	Available FFPE samples	DFS (months)	OS (months)	Tumor regression grade	Recurrence
2	Non-Responder	Pre- and post-treatment	75.1	75.1	1a	
7	Non-Responder	Pre- and post-treatment	6.5	37.4	1a	Local recurrence
9	Non-Responder	Pre- and post-treatment	20.1	78.9	1a	Local recurrence and liver metastasis
11	Non-Responder	Pre- and post-treatment	74.6	74.6	1a	
12	Non-Responder	Pre- and post-treatment	68.3	68.3	1a	
14	Non-Responder	Pre- and post-treatment	57.3	57.3	1a	
17	Non-Responder	Pre- and post-treatment	54.5	54.5	1a	
18	Non-Responder	Pre- and post-treatment	51.3	51.3	1a	
19	Non-Responder	Pre- and post-treatment	49.1	51.5	1a	
8	Non-Responder	Pre-treatment	81.8	81.8	1a	
13	Responder	Pre-treatment	62.5	62.5	2	
1	Responder	Pre-treatment	97.2	97.2	2	
3	Responder	Pre-treatment	99.7	99.7	2	
4	Responder	Pre-treatment	84.3	84.3	2	
5	Responder	Pre-treatment	25.7	85.6	2	
6	Responder	Pre-treatment	82.9	82.9	2	
10	Responder	Pre-treatment	76.9	76.9	2	
15	Responder	Pre-treatment	60.4	60.4	2	
16	Responder	Pre-treatment	60.2	60.2	2	
20	Responder	Pre-treatment	23.7	23.7	2	

CRT, chemoradiotherapy; DFS, Disease-free survival; OS, overall survival.

Supplementary Table S3. The expression of major tumor suppressor genes (PTEN, STK11 and TP53) in the paired tumor samples

	Gene expression (relative coverage)					
	PTEN		*STK11*		*TP53*	
**No**	**Pre**	**Post**	**Pre**	**Post**	**Pre**	**Post**
2	11167	69554	8152	2617	5776	6105
7	41752	67044	15	2085	4182	2445
11	3770	41848	26	3961	0	8926
12	47274	23924	4676	3786	3482	7052
14	25089	32819	3423	4493	1878	7879
17	31429	31944	2406	6017	4314	10663
18	29374	14006	3443	2846	1776	9247

Supplementary Table S4. The immunostaining of p53 in the paired non-responder samples

		Pre (biopsy)			Post (surgical resection)			
**No**	**Grade**	**Area (%)**	**Intensity**	**Score***	**Area (%)**	**Intensity**	**Score***	**Change**
2	1a	30-40	2+	70.0	80	2+	160	increased
7	1a	50	2+	100.0	50	2+	100	no change
9	1a	0	0	0.0	50	1+	50	increased
11	1a	50	1+ ~ 2+	75.0	80	2+	160	increased
12	1a	50	1+ ~ 2+	75.0	50	1+~2+	75	no change
14	1a	30-40	2+	70.0	50	2+	100	increased
17	1a	80	2+	160.0	80	2+	160	no change
18	1a	70	2+	140.0	90	2+	180	increased
19	1a	50-60	1+	55.0	90	2+	180	increased

Score*: (average % of area) x (average intensity)

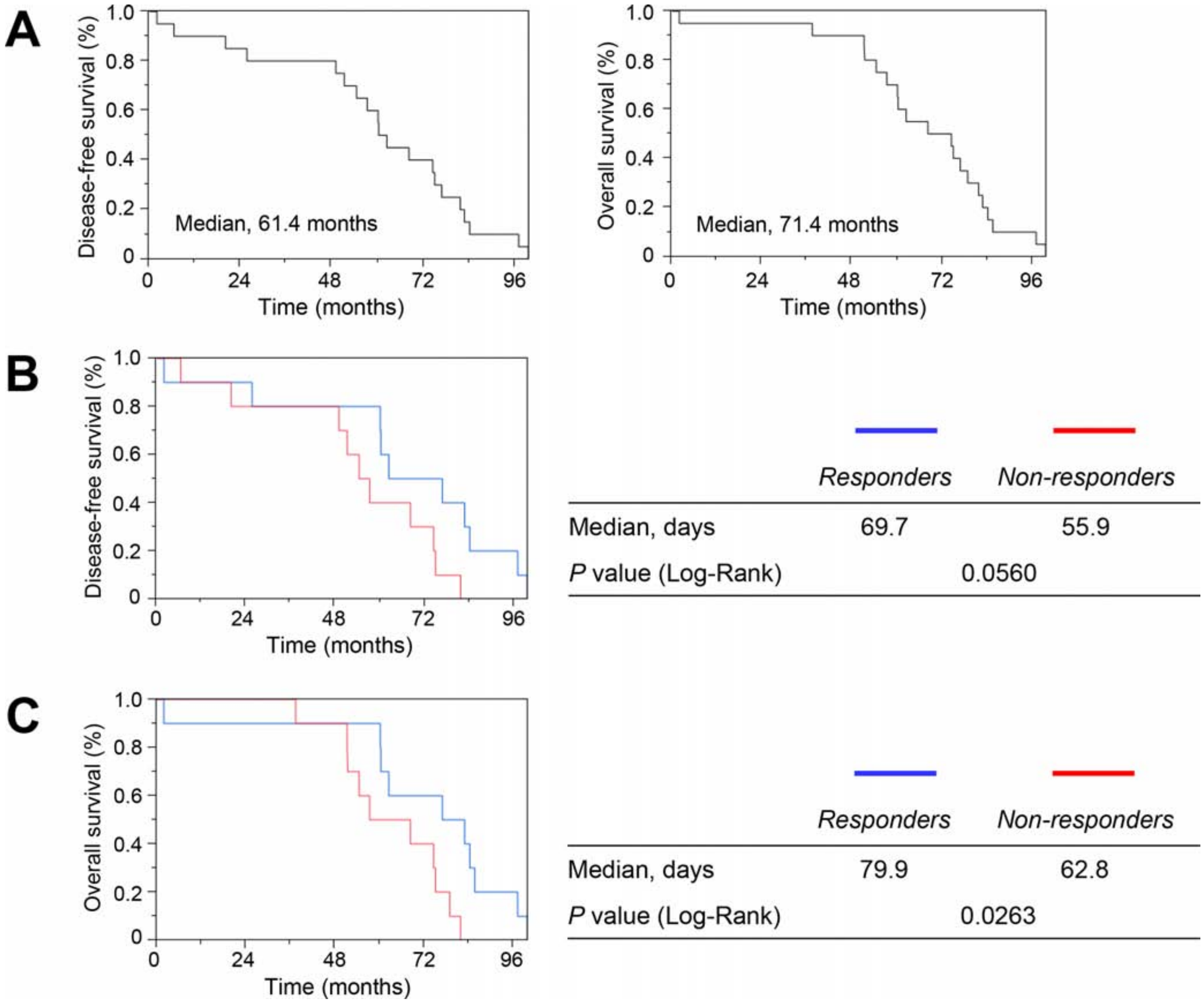

Supplementary Figure S1: Kaplan-Meier curves of disease-free survival (DFS) and overall survival (OS). The survival curves were derived using the Kaplan-Meier method and were compared using log-rank test. A. The median DFS and OS of total patient were 61.4 and 71.4 months, respectively. B. Median DFS in responders versus non-responders was 69.7 versus 55.9 months ($P = 0.0560$). C. Median OS in responders versus non-responders was 79.9 versus 62.8 months ($P = 0.0263$).

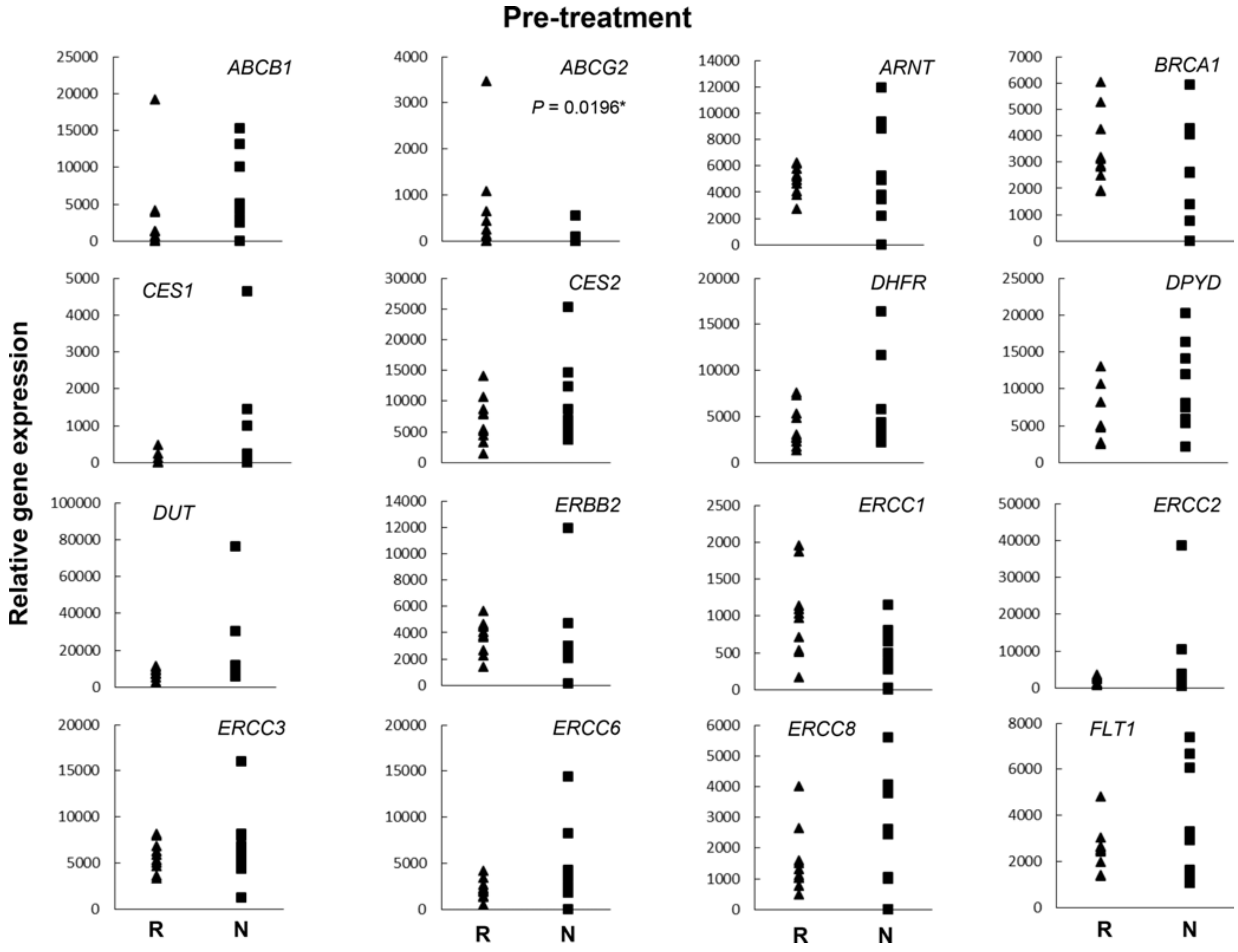

Pre-treatment
Relative gene expression
ABCB1
ABCG2
P = 0.0196*
ARNT
BRCA1
CES1
CES2
DHFR
DPYD
DUT
ERBB2
ERCC1
ERCC2
ERCC3
ERCC6
ERCC8
FLT1
R
N

(*Continued*)

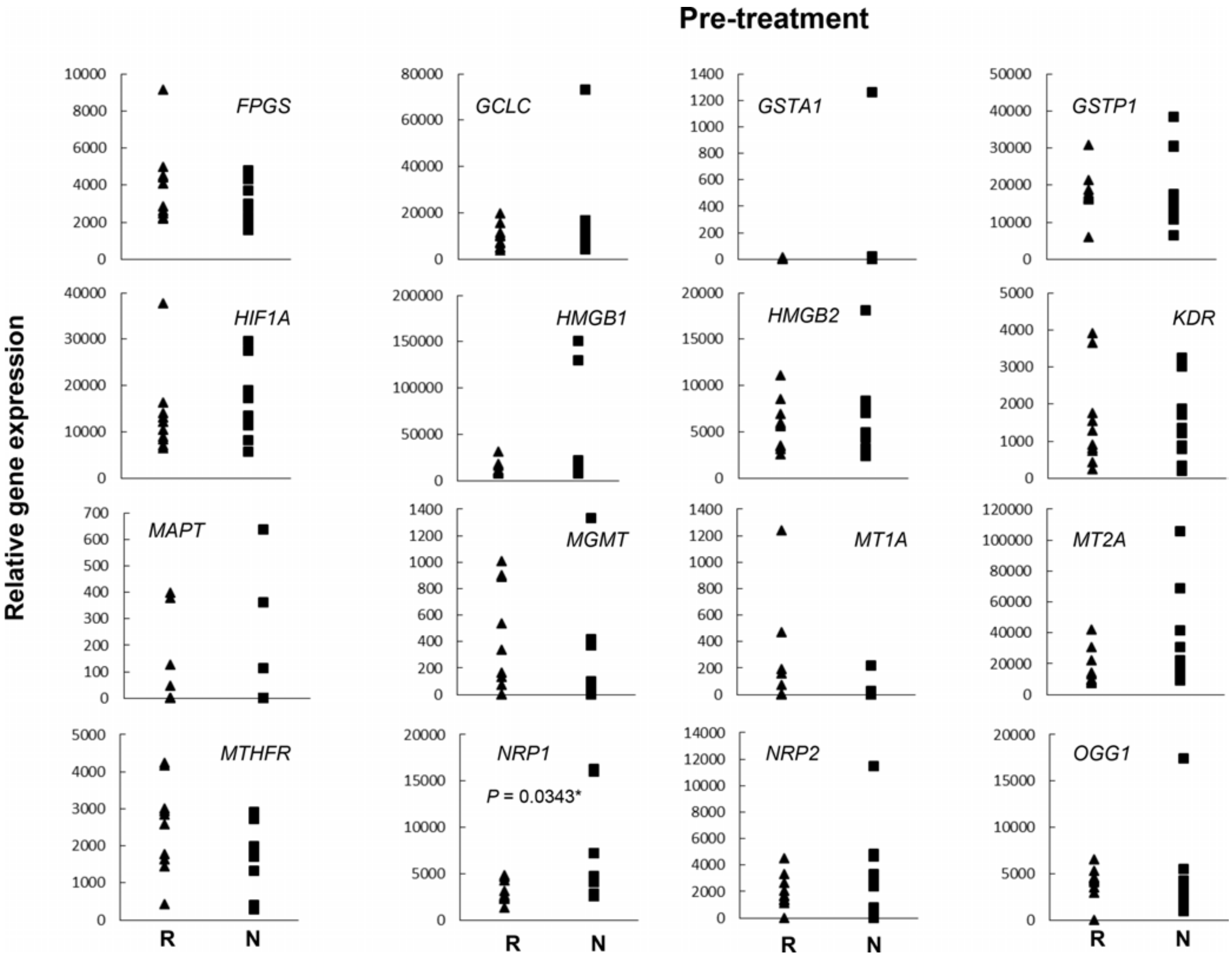
Pre-treatment
Relative gene expression
FPGS
GCLC
GSTA1
GSTP1
HIF1A
HMGB1
HMGB2
KDR
MAPT
MGMT
MT1A
MT2A
MTHFR
NRP1
P = 0.0343*
NRP2
OGG1
R
N

(*Continued*)

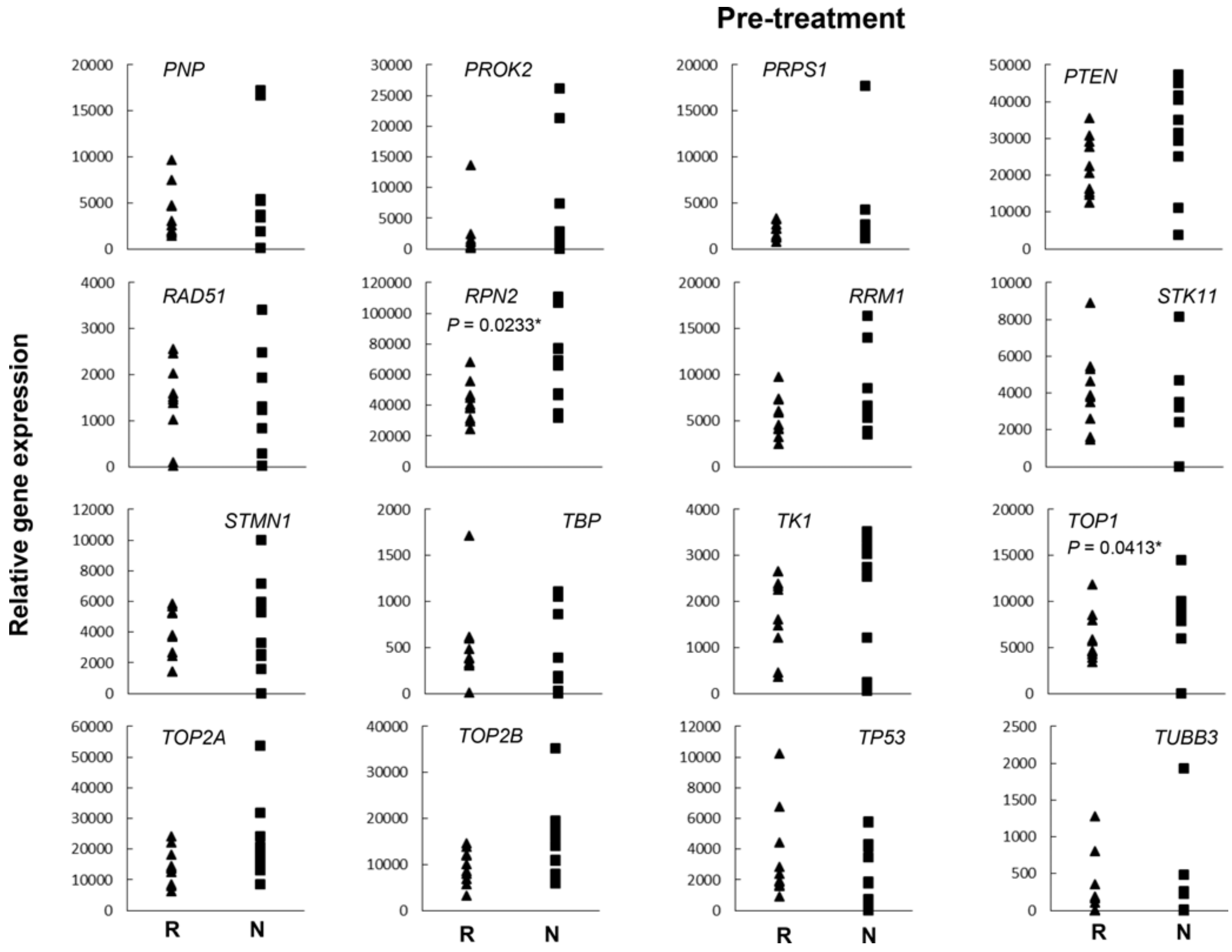
Pre-treatment
Relative gene expression
PNP
PROK2
PRPS1
PTEN
RAD51
RPN2
P = 0.0233*
RRM1
STK11
STMN1
TBP
TK1
TOP1
P = 0.0413*
TOP2A
TOP2B
TP53
TUBB3
R
N

(*Continued*)

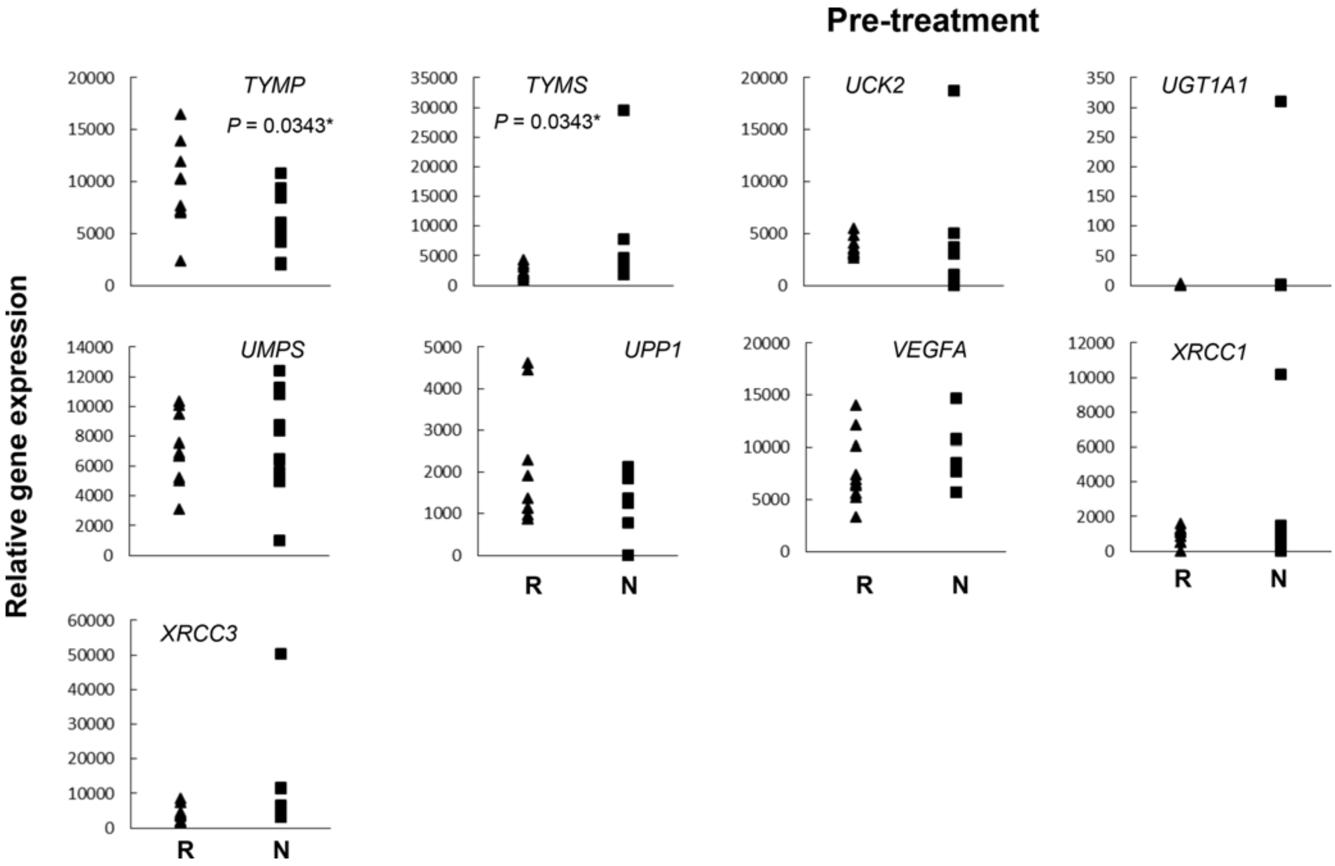

Supplementary Figure S2: The changes in the relative gene expression between responder and non-responder samples. A non-parametric statistical method (Mann-Whitney U tests) was used for the statistical analysis. R, responder; N, non-responder

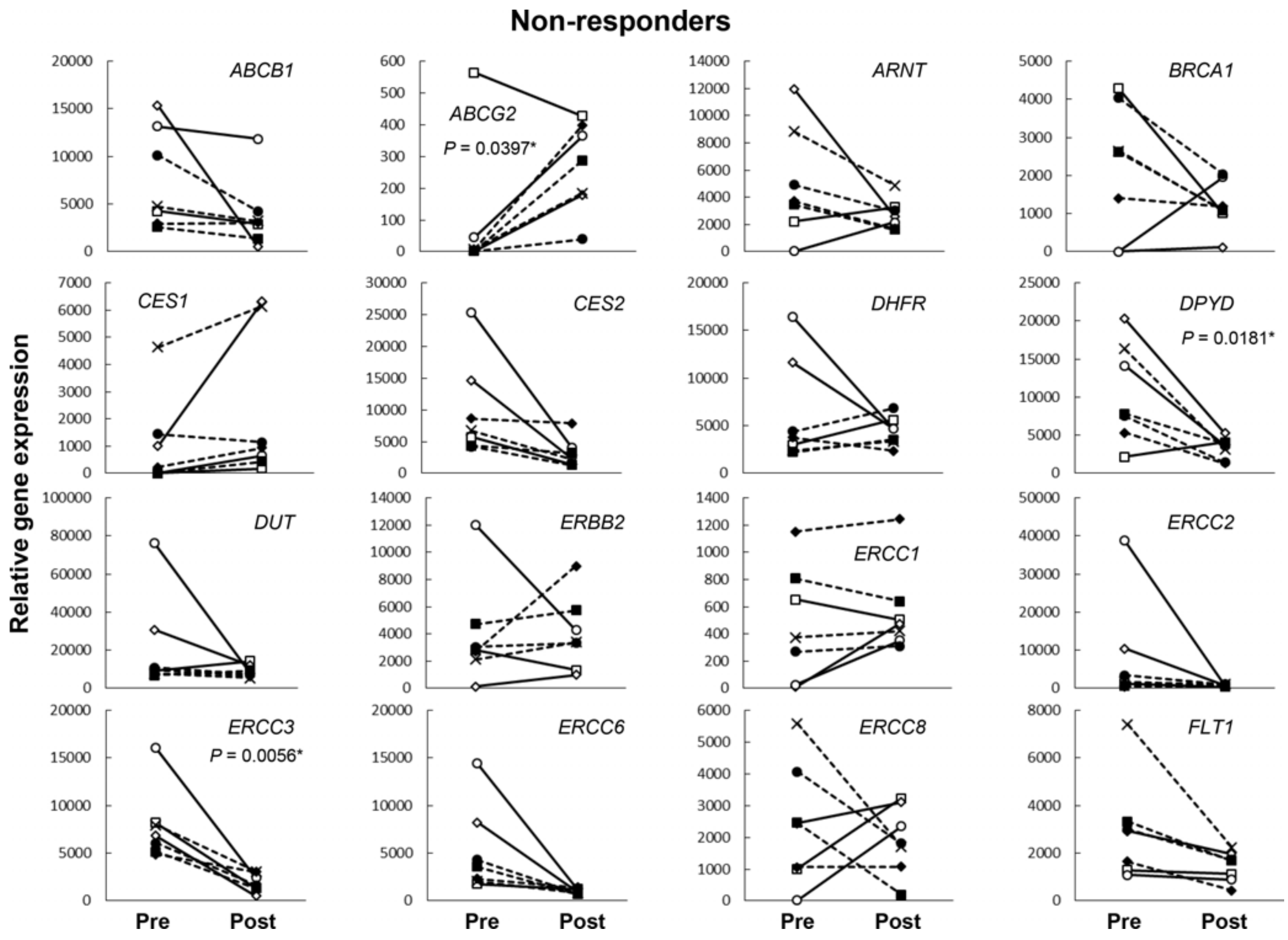
Non-responders
Relative gene expression
ABCB1
ABCG2
P = 0.0397*
ARNT
BRCA1
CES1
CES2
DHFR
DPYD
P = 0.0181*
DUT
ERBB2
ERCC1
ERCC2
ERCC3
P = 0.0056*
ERCC6
ERCC8
FLT1
Pre
Post

(*Continued*)

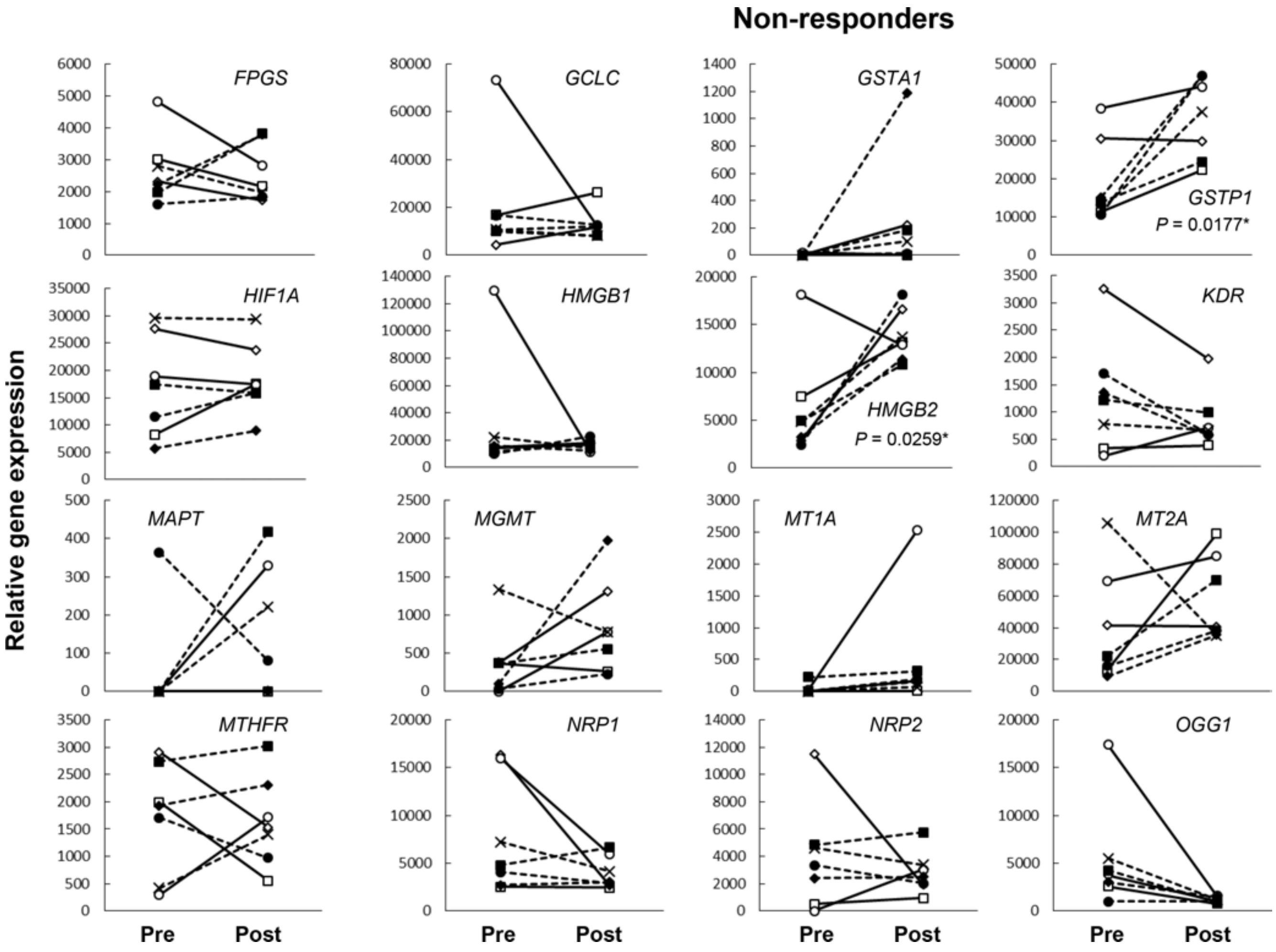
Non-responders
Relative gene expression
FPGS
GCLC
GSTA1
GSTP1
P = 0.0177*
HIF1A
HMGB1
HMGB2
P = 0.0259*
KDR
MAPT
MGMT
MT1A
MT2A
MTHFR
NRP1
NRP2
OGG1
Pre
Post

(*Continued*)

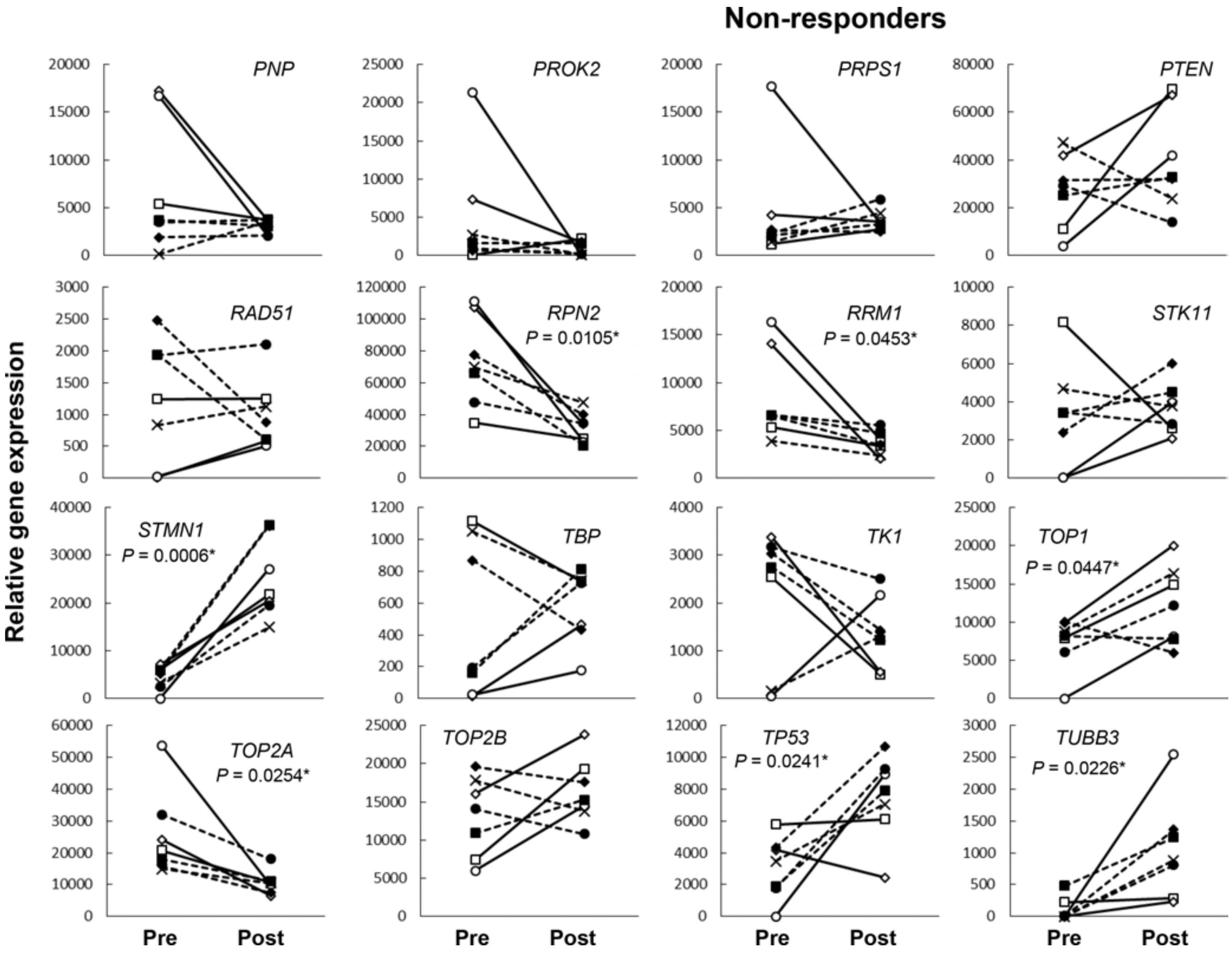
Non-responders
Relative gene expression
PNP
PROK2
PRPS1
PTEN
RAD51
RPN2
P = 0.0105*
RRM1
P = 0.0453*
STK11
STMN1
P = 0.0006*
TBP
TK1
TOP1
P = 0.0447*
TOP2A
P = 0.0254*
TOP2B
TP53
P = 0.0241*
TUBB3
P = 0.0226*
Pre
Post

(*Continued*)

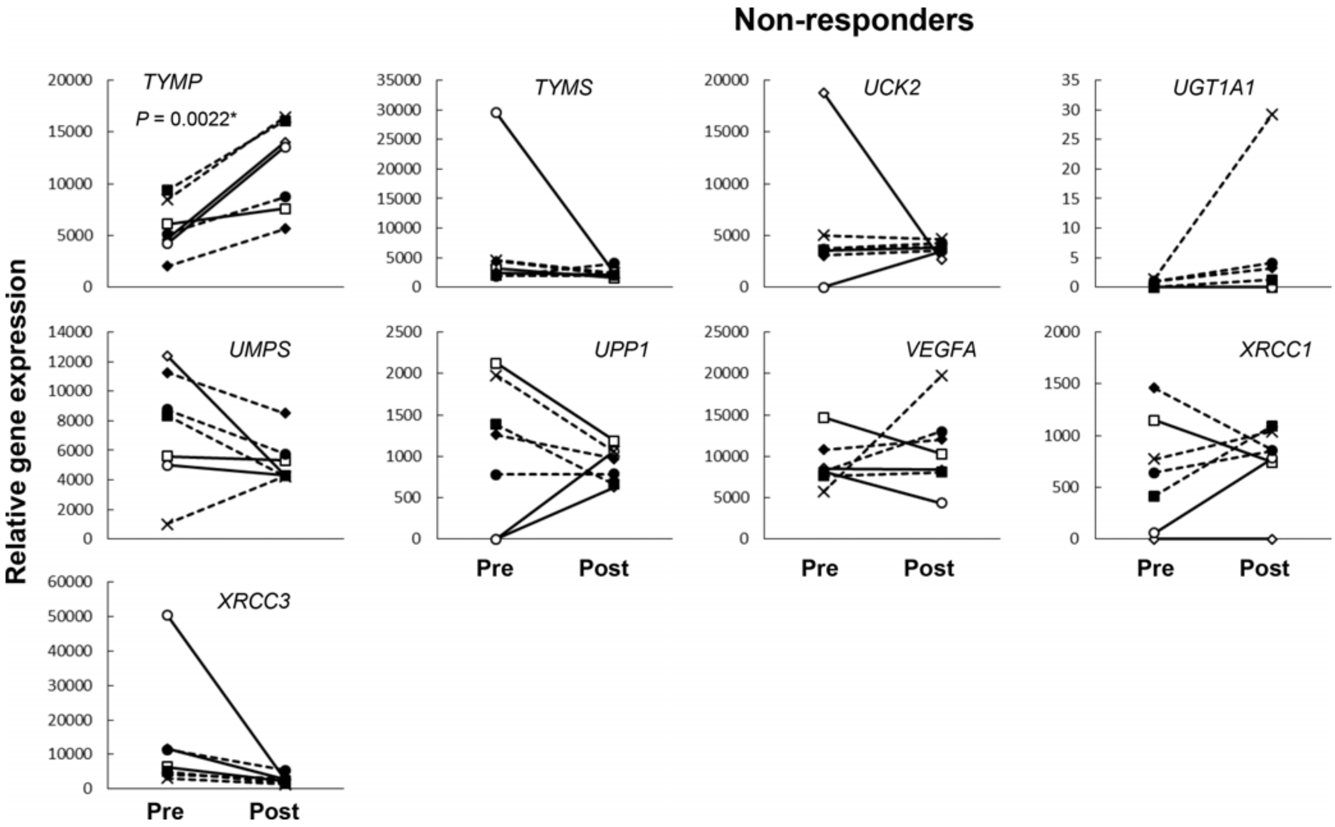

Supplementary Figure S3: The changes in the relative gene expression for paired non-responder tumor samples. Paired t-tests were used for the statistical analysis.
